# Supplementary material for: Statin use and outcome risks according to predicted CVD risk in Korea: A retrospective cohort study
Source: PLoS One. 2021 Jan 15;16(1):e0245609. doi: 10.1371/journal.pone.0245609 (PMC7810517; doi:10.1371/journal.pone.0245609)
Supplement: S1 File — (DOCX) [file pone.0245609.s002.docx]

**Statin use and outcome risks according to predicted CVD risk in Korea: A retrospective cohort study**

**Contents**

**S1 Text.** Baseline Covariates

**S2 Text.** Risk Categories

**S3 Text.** NHIS Sample Cohort of Health Screening 2002–2015

**S1 Fig.** Hazard Ratios for CVD and Non-CVD Outcomes in a NHIS-Generated Sample Cohort

**S2 Fig.** Absolute Event Rates According to the 10-Year Risk of CVD or the Presence of Risk Factors

**S1 Table.** Definitions of Exposures, Risk Factors, and Outcomes

**S2 Table.** Medications and In-Hospital Procedures Retrieved From NHIS Reimbursement Records

**S3 Table.** Participation Rates in Health Screenings During The Study Periods

**S4 Table.** ICD-10 Diagnosis Codes

**S5 Table.** Time-Lagged Covariates for Subsequent Years

**References**

**S1 Text. Baseline Covariates**

Baseline data were obtained from the records of nationwide health screenings performed between 2006 and 2011, generally at 2-year intervals (S3 Table). The covariates were determined using the averaged values for 2006–2011, except for the values of HDL cholesterol and eGFR, which were averaged for 2009–2011.

Outlier data were deleted on the basis of the ranges required in current CVD risk calculators [1].

(a) total cholesterol <130 mg/dl or >320 mg/dl

(b) HDL cholesterol <20 mg/dl or >100 mg/dl

(c) systolic BP <90 mm Hg or >200 mm Hg

The data of extreme values, which might be resulted from recording mistakes, were also deleted.

(a) fasting blood glucose <30 mg/dl or >900 mg/dl

(b) serum creatinine <0.3 mg/dl or >15.0 mg/dl

(c) body mass index <10.0 kg/m^2^ or >50.0 kg/m^2^

After deleting missing or outlier data of the variables, statin-treated and untreated total cholesterol records were collected separately. Statin-treated cholesterols were identified as the cases that received statins for ≥90 days in the year of measurement, and untreated cholesterols as the cases that received statins for <90 days in that year. For a missing untreated total cholesterol, the product of treated total cholesterol and 1.10 (the mean ratio of untreated to treated levels in participants in whom both levels were available) was imputed. Then, untreated total cholesterol was categorized into 5 groups based on the ATP III classifications [2]. HDL cholesterol was categorized into 3 groups,.

Systolic blood pressure was categorized into seven 10-mm Hg blood pressure groups incorporating the cutoff values of prehypertension and hypertension. Fasting blood glucose was categorized into 7 groups primarily with 20 mg/dl intervals and additionally incorporating the cutoff values for prediabetes and diabetes [3]. Body mass index was categorized into 5 groups (underweight, 10.0–18.4; low-normal weight, 18.5–22.9; high-normal weight, 23.0–24.9, overweight, 25.0–29.9; and obesity, 30.0–50.0 kg/m^2^) referring to the WHO classifications [4].

Proteinuria was identified as urine dipstick albuminuria of ≥1+ once or ≥trace at least twice during health screenings between 2006 and 2011. The eGFR was calculated using the CKD-EPI creatinine equation and was categorized into 4 groups according to the KDIGO guidelines [5].

Baseline age was classified into 5-year age categories. Sex and family history of CVD were categorized dichomonously. Drinking amount was calculated as the number of drinks averaged per day and was categorized into 5 groups. Income levels were determined by income based insurance contributions. The vigintile values of insurance contributions were averaged from 2006 to 2011. The averaged values were categorized into 3 groups (0.0–7.9, 8.0–14.9, or 15.0–20.0).

**S2 Text. Risk Categories**

Participants were categorized according to the 10-year risk of CVD (≥10%, 5.0–9.9%, or <5.0%) predicted using the 2018 revised Pooled Cohort Equations [6].

Participants were also categorized by the presence of risk factors (≥3, 2, or ≤1 risk factors) at baseline.

**A-1. The 10-year risk of CVD events was calculated with the following variables.**

(a) Age: age (year) in 2011

(b) T_SBP: time averaged values of treated systolic BP (mm Hg) for 2006–2011

(b) U_SBP: time averaged values of untreated systolic BP (mm Hg) for 2006–2011

(d) Total_C: time averaged values of untreated total cholesterol (mg/dl) for 2006–2011

(e) HDL_C: time averaged values of HDL cholesterol (mg/dl) for 2009–2011

(f) Cig: smoking status (yes or no) on January 1, 2012

(g) DM: diabetes status (yes or no) on January 1, 2012

**A-2. Calculation of CVD risk scores using the revised Pooled Cohort Equations.**

In males treated with antihypertensive drugs

CRISK

= -11.679980

+ (0.064200 * Age)

+ (-0.000061 * T_SBP**2)

+ (0.038950 * T_SBP)

+ (2.055533)

+ (0.842209 *DM)

+ (0.895589 * Cig)

+ (0.193307 * Total_C / HDL_C)

+ (-0.014207 * T_SBP)

+ (0.000025 * Age * T_SBP);

In male nonusers of antihypertensive drugs

CRISK

= -11.679980

+ (0.064200 * Age)

+ (-0.000061 * U_SBP**2)

+ (0.038950 * U_SBP)

+ (0.842209 *DM)

+ (0.895589 * Cig)

+ (0.193307 * Total_C / HDL_C)

+ (0.000025 * Age * U_SBP);

In males

10-year risk of CVD = 100 / (1 + exp(-CRISK));

In females treated with antihypertensive drugs

CRISK

= -12.823110

+ (0.106501 * Age)

+ (0.000056 * T_SBP**2)

+ (0.017666 * T_SBP)

+ (0.731678)

+ (0.943970 *DM)

+ (1.009790 * Cig)

+ (0.151318 * Total_C / HDL_C)

+ (-0.003647 * T_SBP)

+ (-0.000153 * Age * T_SBP);

In female nonusers of antihypertensive drugs

CRISK

= -12.823110

+ (0.106501 * Age)

+ (0.000056 * U_SBP**2)

+ (0.017666 * U_SBP)

+ (0.943970 *DM)

+ (1.009790 * Cig)

+ (0.151318 * Total_C / HDL_C)

+ (-0.000153 * Age * U_SBP);

In females

10-year risk of CVD = 100 / (1 + exp(-CRISK));

**B. Determination of risk factors**

Five risk factors (hypertension, diabetes mellitus, dyslipidemia, proteinuria, and active smoking; S1 Table) were identified, using the results of health screenings and information on the prescription of drugs. Information on the prescription of drugs (S2 Table) in the reimbursement records were retrieved using NHIS billing codes.

**S3 Text. NHIS Sample Cohort of Health Screening 2002–2015**

The hazard ratios for adverse outcomes were reanalyzed in a National Health Insurance Service (NHIS)-generated sample cohort [7].

A total of 514,866 adults were randomly selected from 5.15 million citizens aged 40–79 years who underwent health screening in 2002 or 2003 and were followed-up through December 31, 2015. Of the 514,866 adults, 19,265 with missing or outlier data; 29,329 with a history of heart disease, stroke, or cancer or were diagnosed with chronic kidney disease; and 36,479 who died or developed cardiovascular disease (CVD), major cancers, or end-stage kidney disease before baseline (i.e., January 1, 2008) were excluded. Among the remaining 429,793 adults, 24,314 users who initiated statins during the 2003–2006 period and 388,935 nonusers who never or rarely used statins before baseline were identified. In the sample of 413,249 adults, the propensity scores for statin users were calculated using a logistic regression with a set of measured covariates. The pairs of statin users and nonusers were created employing a greedy, nearest-neighbor matching algorithm with a caliper width of 0.2.

The propensity score matched cohort finally included 23,912 pairs of users and nonusers of statins. The matched groups had balanced baseline characteristics with standardized differences less than 0.10.

In the propensity score matched cohort, multivariable-adjusted hazard ratios were estimated using Cox models with time-varying covariates. The yearly updated statin use status and the yearly antihypertensive and antidiabetic use status were entered as time-varying covariates . The baseline levels of age, sex, family history of CVD, income, physical exercise, drinking, smoking, body mass index, systolic blood pressure, blood glucose, untreated total cholesterol, and proteinuria were entered as fixed covariates (the baseline HDL cholesterol or estimated glomerular filtration rate was not available in this cohort).

In a total of 24,314 users who initiated statins in 2003–2006, the hazard ratios in regular statin users were estimated in comparison with occasional rather than nonusers. The model included the yearly medication use status as time-varying covariates and the baseline covariates along with the year of statin initiation as fixed covariates.


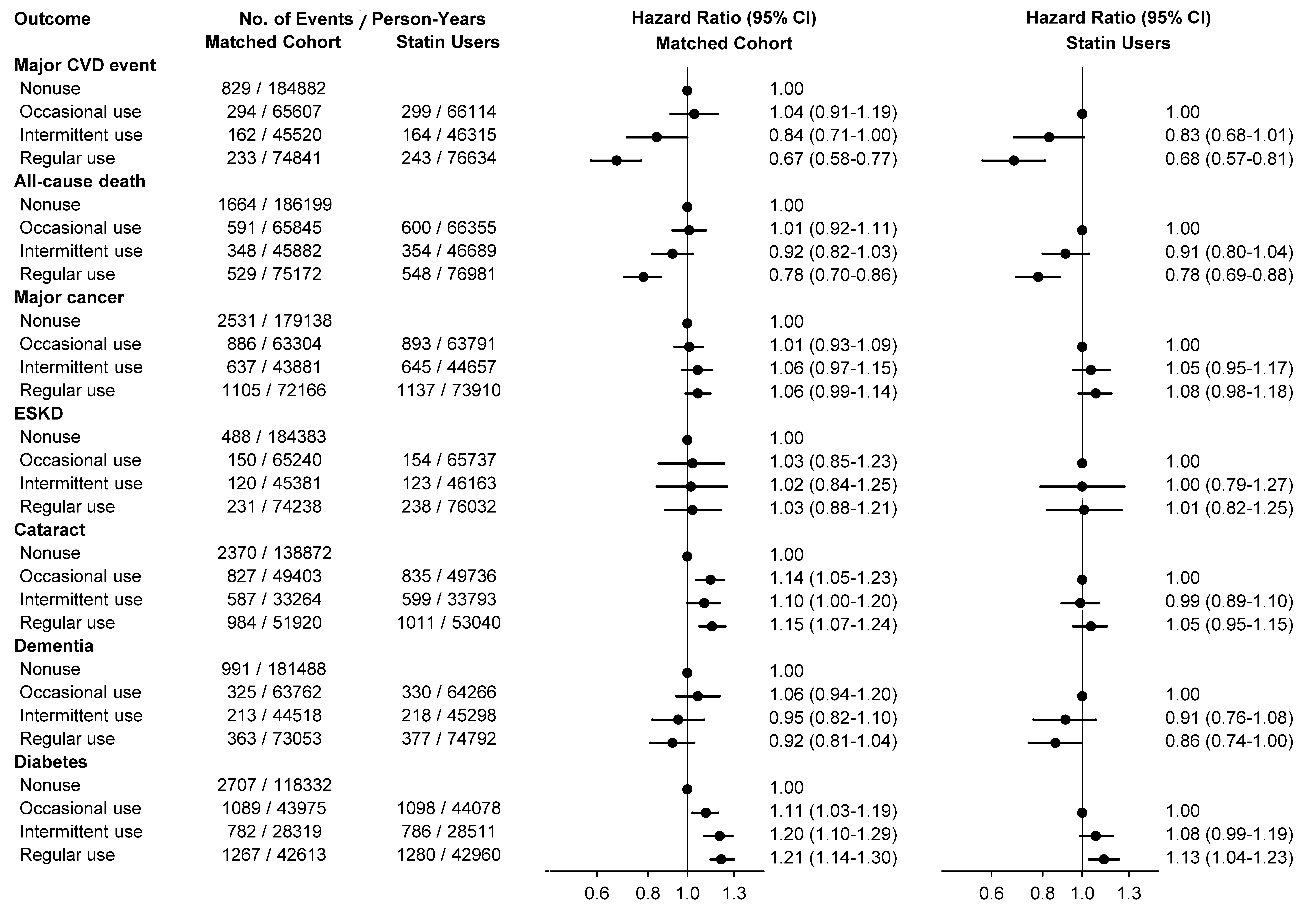


**S1 Fig. Hazard Ratios for CVD and Non-CVD Outcomes in a NHIS-Generated Sample Cohort**

Hazard ratios were estimated using Cox models with a time-varying covariate for statin use status. In the analyses of 23,912 pairs matched on propensity scores, baseline nonusers served as the reference. In secondary analyses within 24,314 statin users, occasional users served as the reference. All hazard ratios were adjusted for time-varying covariates of yearly updated antihypertensive and antidiabetic use status and for fixed covariates of baseline age, sex, family history of CVD, income, physical exercise, drinking, smoking, body mass index, systolic blood pressure, fasting blood glucose, untreated total cholesterol, and proteinuria (and the year of statin initiation in the secondary analyses). The participants who were diagnosed with cataract (or dementia, or diabetes) before the baseline were excluded from the analysis of cataract (or dementia, or diabetes). CVD, cardiovascular disease; ESKD, end-stage kidney disease; NHIS, National Health Information Database.


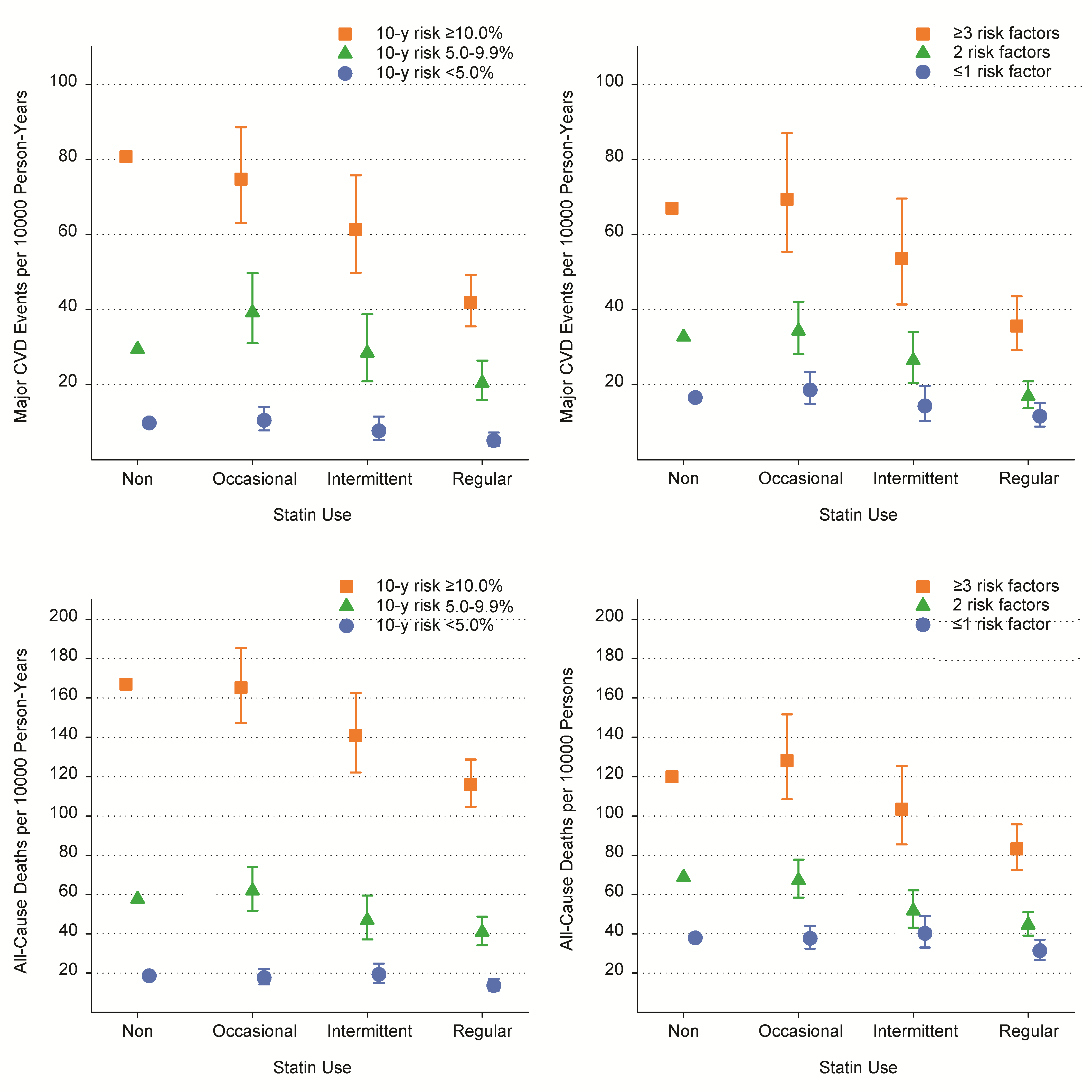


**S2 Fig. Absolute Event Rates According to the 10-Year Risk of CVD or the Presence of Risk Factors**

The absolute event rates and 95% CIs were calculated by multiplying the hazard ratios and 95% CIs by the rates observed within the nonuser group. Multivariable-adjusted hazard ratios were estimated using Cox models with a time-varying covariate for statin use status, in subgroups stratified by the 10-year risk of CVD or the presence of risk factors. CVD, cardiovascular disease.

**S1 Table. Definitions of Exposures, Risk Factors, and Outcomes**

| **Condition** | **Definition** |
| --- | --- |
| **Statin use status** |  |
| Regular use | prescription of statins for >2/3 of each period of statin therapy |
| Intermittent use | prescription of statins for 1/3–2/3 of each period of statin therapy |
| Occasional use | prescription of statins for <1/3 of each period and ever for ≥90 days per year |
| Nonuse | prescription of statins never for ≥90 days per year |
| **Risk factor** |  |
| Hypertension | BP ≥140/90 mm Hg or prescription of antihypertensives for ≥90 days per year |
| Diabetes | fasting glucose ≥126 mg/dl or prescription of antidiabetics for ≥90 days per year |
| Dyslipidemia | untreated total cholesterol ≥240 mg/dl or HDL cholesterol <40 mg/dl |
| Proteinuria | urine dipstick albumin ≥1+ once or ≥trace at least twice |
| Active smoking | current cigarette smoking |
| **Outcome** |  |
| Major CVD event | revascularization or CCU admission for myocardial infarction, revascularization or CCU admission for stroke, or death from CVD |
| All-cause mortality | death from any cause |
| Diabetes | fasting glucose ≥126 mg/d or prescription of antidiabetics for ≥90 days per year |
| Severe cataract | cases that underwent surgery for cataract |
| Dementia | cases that received memantine or cholinesterase inhibitors for dementia |
| ESKD | dialysis for 90 or more days per year or kidney transplantation |
| Major cancers | lung cancer, hepatoma, colon cancer, stomach cancer, pancreatic cancer, gallbladder and bile duct cancer, or breast cancer |

CCU, critical care unit; CVD, cardiovascular disease; ESKD, end-stage kidney disease.

**S2 Table. Medications and In-Hospital Procedures Retrieved From NHIS Reimbursement Records**

| **Statin** | Atorvastatin, Rosuvastatin, Simvastatin, Pravastatin, Pitavastatin, Fluvastatin, Lovastatin, Cerivastatin |
| --- | --- |
| **Antihypertensive** | Thiazide, β blocker, α antagonist, Central sympatholytic, ACE inhibitor, AG II antagonist, Calcium antagonist, Direct vasodilator |
| **Antidiabetic** | Metformin, Sulfonylurea, Meglitinide, Glitazone, DPP-4 inhibitor, SGLT2 inhibitor, GLP-1 agonist, α-glucosidase inhibitor, Insulin |
| **Coronary revascularization** | Percutaneous coronary angioplasty, Percutaneous coronary stent insertion, Percutaneous coronary thrombolysis or thrombectomy, Percutaneous coronary atherectomy, Coronary artery bypass graft, Coronary artery endarterectomy |
| **Cerebral or carotid revascularization** | Percutaneous cerebral angioplasty, Percutaneous cerebral stent insertion, Percutaneous cerebral thrombolysis or thrombectomy, Percutaneous carotid angioplasty, Percutaneous carotid stent insertion, Percutaneous carotid thrombolysis or thrombectomy, Transluminal carotid atherectomy, Carotid endarterectomy |
| **CCU admission** | Admission to CCU |
| **Antidementia drug** | Donepezil, Rivastigmine, Galantamine, Memantine |
| **Cataract surgery** | Surgery for cataract, Phacoemulsification, Primary intraocular lens implantation |
| **Renal replacement therapy** | Hemodialysis, Kidney transplantation |
| **Peritoneal Dialysate** | Peritosol |

ACE, angiotensin-converting-enzyme; AG, angiotensin; CCU, critical care unit; DPP-4 dipeptidyl peptidase-4; GLP-1, glucagon-like peptide-1; NHIS, National Health Insurance Service; SGLT2, sodium-glucose transport protein 2.

**S3 Table. Participation Rates in Health Screenings During The Study Periods**

| **Period, year** | **Survivors at The End of Period, no.** | **Participants in Health Screening, no. (%)** |
| --- | --- | --- |
| 2006–2007 | 116,530 | 72,582 (62.3%) |
| 2009–2009 | 116,530 | 116,530 (100%) |
| 2011–2011 | 116,530 | 90,485 (77.6%) |
| 2013–2013 | 115,551 | 91,297 (79.0%) |
| 2014–2015 | 114,178 | 88,977 (77.9%) |
| 2016–2017 | 112,487 | 85,058 (75.6%) |

**S4 Table. ICD-10 Diagnosis Codes**

| **Diagnosis** | **ICD-10 Code** |
| --- | --- |
| Myocardial infarction | I21, I22 |
| Ischemic stroke | I63, I64 |
| CVD | I00–I99 |
| Dementia | F00–F03 |
| Cataract | H25, H26, H28 |
| Kidney transplantation | Z94.0 |
| Major cancer | lung C34; liver C22; colon C18–C21; stomach C16; pancreas C25; gallbladder and bile duct C23, C24; breast C50 |

CVD, cardiovascular disease; ICD-10, International Classification of Diseases-10th Revision.

**S5 Table. Time-Lagged Covariates for Subsequent Years**

| **Year of Outcome Variable,**  **Clinical Endpoint^†^** | **Year of Time-Varying Covariate,**  **Medication Use Status**^‡^ | **Period of Fixed Covariate,**  **Baseline Value**^§^ |
| --- | --- | --- |
|  |  |  |
| 2012 | 2011 | 2006–2011 |
| 2013 | 2012 | 2006–2011 |
| 2014 | 2013 | 2006–2011 |
| 2015 | 2014 | 2006–2011 |
| 2016 | 2015 | 2006–2011 |
| 2017 | 2016 | 2006–2011 |

^†^ All-cause death, major CVD events, diabetes mellitus, dementia, cataract, ESKD, and major cancers.

^‡^ Statin, antihypertensive, and antidiabetic use statuses.

^§^ Age, sex, family history of CVD, income, physical exercise, drinking, body mass index, systolic blood pressure, total cholesterol, HDL cholesterol, proteinuria, and eGFR.

CVD, cardiovascular disease; ESKD, end-stage kidney disease; eGFR, estimated glomerular filtration rate; HDL, high-density lipoprotein.

**References**

1. ACC/AHA ASCVD Risk Calculator. [cited 22 Aug 2020]. Available: http://www.cvriskcalculator.com/

2. Expert Panel on Detection E and Treatment of High Blood Cholesterol in Adults. Executive Summary of The Third Report of The National Cholesterol Education Program (NCEP) Expert Panel on Detection, Evaluation, And Treatment of High Blood Cholesterol In Adults (Adult Treatment Panel III). JAMA. 2001;285: 2486–2497.

3. American Diabetes Association. 2. Classification and Diagnosis of Diabetes. Diabetes Care. 2016;39 Suppl 1: S13-22. doi:10.2337/dc16-S005

4. WHO Expert Consultation. Appropriate body-mass index for Asian populations and its implications for policy and intervention strategies. Lancet. 2004;363: 157–163. doi:10.1016/S0140-6736(03)15268-3

5. Stevens PE, Levin A, Kidney Disease: Improving Global Outcomes Chronic Kidney Disease Guideline Development Work Group Members. Evaluation and management of chronic kidney disease: synopsis of the kidney disease: improving global outcomes 2012 clinical practice guideline. Ann Intern Med. 2013;158: 825–830. doi:10.7326/0003-4819-158-11-201306040-00007

6. Yadlowsky S, Hayward RA, Sussman JB, McClelland RL, Min Y-I, Basu S. Clinical Implications of Revised Pooled Cohort Equations for Estimating Atherosclerotic Cardiovascular Disease Risk. Ann Intern Med. 2018;169: 20–29. doi:10.7326/M17-3011

7. Lee J, Lee JS, Park S-H, Shin SA, Kim K. Cohort Profile: The National Health Insurance Service-National Sample Cohort (NHIS-NSC), South Korea. Int J Epidemiol. 2017;46: e15. doi:10.1093/ije/dyv319
