# Supplementary material for: Statin use and outcome risks according to predicted CVD risk in Korea: A retrospective cohort study
Source: PLoS One. 2021 Jan 15;16(1):e0245609. doi: 10.1371/journal.pone.0245609 (PMC7810517; doi:10.1371/journal.pone.0245609)
Supplement: Attachment — Submitted filename: author_response_letter.docx [file pone.0245609.s003.docx]

Manuscript ID: PMEDICINE-D-20-00850

I thank you and the reviewers for your thoughtful comments and suggestions. The manuscript has benefited from the comments.

The manuscript has been rechecked and the necessary changes have been made in accordance with the reviewers’ suggestions. The responses to all comments have been prepared and attached herewith, in addition to having been implemented in the manuscript, as indicated.

Requests from the editors:

*Please revise your title according to PLOS Medicine's style - this should sum up (in the first part of the title) the study question, followed by the study design, after a colon (eg, "A randomized controlled trial," "A retrospective study," "A modelling study"). Please note we saw that one reviewer commented the authors have done a retrospective cohort while another states this is prospective - the authors should be clear on the study design.

Response: The Title has been revised to “Statin use and outcome risks according to predicted CVD risk in Korea: A retrospective cohort study”. The study was conducted using the public database that had collected data for general research purposes rather than specifically for this study.

*In the last sentence of the Abstract Methods and Findings section, please include a brief note about any key limitation(s) of the study's methodology.

Response: I have revised the Abstract to include key limitations of this study.

*At this stage, we ask that you include a short, non-technical Author Summary of your research to make findings accessible to a wide audience that includes both scientists and non-scientists. The Author Summary should immediately follow the Abstract in your revised manuscript. This text is subject to editorial change and should be distinct from the scientific abstract. Please see our author guidelines for more information: https://journals.plos.org/plosmedicine/s/revising-your-manuscript#loc-author-summary

Response: I have revised the manuscript to include the Author summary.

*Please clarify in the paper if the analysis reported here corresponds to one laid out in a prospectively set out protocol or analysis plan? Please state this (either way) early in the Methods section.

Response: I have stated that in the 1st paragraph in the Methods section.

a) If a prospective analysis plan (from your funding proposal, IRB or other ethics committee submission, study protocol, or other planning document written before analyzing the data) was used in designing the study, please include the relevant prospectively written document with your revised manuscript as a Supporting Information file to be published alongside your study, and cite it in the Methods section. A legend for this file should be included at the end of your manuscript.

Response: There was no documented protocol or proposal except for IRB submission.

b) If no such document exists, please make sure that the Methods section transparently describes when analyses were planned, and when/why any data-driven changes to analyses took place.

Response: In the first paragraph in the Methods, I documented IRB File Number that included the information on the date of approval and described when the analyses were conducted.

c) In either case, changes in the analysis-- including those made in response to peer review comments-- should be identified as such in the Methods section of the paper, with rationale.

Response: In the 9th paragraph in the Methods, I have mentioned the additional analysis made in response to peer review comments.

*We'd suggest using an appropriate reporting guideline, eg the STROBE guideline (https://www.equator-network.org/reporting-guidelines/strobe/) to support reporting of this study. In this case, please add the following statement, or similar, to the Methods: "This study is reported as per the Strengthening the Reporting of Observational Studies in Epidemiology (STROBE) guideline (SChecklist)." When completing the checklist, please use section and paragraph numbers, rather than page numbers, and then upload the completed reporting checklist as supporting information with the revised paper.

Response: I have added the statement to the Methods, completed the checklist, and uploaded the checklist.

-----------------------------------------------------------

Comments from the reviewers:

Reviewer #1: I confine my remarks to statistical aspects of this paper. The general approach is fine but I have a couple requests for clarification and a couple issues to resolve before I can recommend publication.

The major issue is not to categorize continuous independent variables. In *Regression Modeling Strategies* Frank Harrell lists 11 problems with this and sums up "nothing could be more disastrous". I wrote a blog piece showing, graphically, the problems that can arise. https://medium.com/@peterflom/what-happens-when-we-categorize-an-independent-variable-in-regression-77d4c5862b6c?source=friends_link&sk=1428cd15968e218268121dc507ce8025

Response: Thank you for raising these important issues. I acknowledge that continuous independent variables can provide more valuable information than the variables categorized. Likewise, time-updated independent variables may have a stronger association with outcome risks than the variables measured at baseline [1].

In accordance with your suggestions, I have additionally performed the analysis in spline regression models. To explore the validity of the Pooled Cohort Equations risk calculator in this Korean cohort of statin users and nonusers, the hazard ratios for cardiovascular disease (CVD) events were estimated with the predicted risk scores using the restricted cubic spline. The results of the spline regression models have been included in the manuscript (Fig 6 and the 5th paragraph in the Discussion). The linear association between the risk scores and the CVD hazard ratios indicates consistent discrimination over the risk scores (althouth it does not ensure individual-level discrimination). Moreover, the similar slopes between statin users and nonusers at least do not oppose the thesis that statin-associated relative risk reduction for CVD is not different across the risk scores. I believe that this analysis has improved the manuscript providing additional valuable information.

However, unfortunately, Cox regression with restricted cubic splines could not readily be performed with time-updated statin use status, in the dataset prepared for the final analysis. If it was performed successfully, more information could be provided. However, I believe that its advantage might not be large for the statin use status that had a limited range (prescription days per medication period days: occasional, 0.00–0.33; intermittent, 0.34–0.66; and regular, 0.67–1.00). In contrast, the risk scores had a wide range of continuous values (low, 0.1–4.9%; intermediate, 5.0–9.9%; and high,10.0–90.0%).

Other issues:

p. 4 - Please give details of what the outlier data were, how outliers were detected and why they were deleted. Deleting outliers simply because they are outliers is a bad idea.

Response: The outlier data were deleted in consideration of the ranges required in current CVD risk calculators [2]. The Supplement has been revised to include information on outliers (S1 Text).

p. 5 near bottom - On p. 4 you said you deleted missing cases. Here you say you did single imputation. Please clarify. Also, unless the proportion of missing data is very small, single imputation is not recommended

Response: I deleted missing cases applying the same criteria for all variables (i.e., , I deleted the cases with no available data for total cholesterols, with no baseline blood pressures, and with no baseline data for other variables, regardless of medication use status). Then, I imputed the untreated total cholesterol levels (if not available) using treated levels. I have revised the Supplement to describe the process more detailedly (S1 Text). Compared with a single deletion of missing untreated total cholesterol, this imputation might not increase but rather decrease the risk of selection bias resulting from uneven exclusion or inclusion of the cases.

p. 6 Risk categories These could be useful for stratified analysis, but not for other purposes. It would be better to give each person a score (from 0 to 100 or whatever)

Response: The ranges in the Risk categories have been revised to include the risk scores of all participants, as you recommended.

p 7 How much lag in the time lagged variables?

Response: Using yearly updated covariates, the analysis assessed the risk of clinical events that occurred in the next years. I have created a new S5 Table to clarify this.

p. 8 Congratulations for using SMD rather than p value. SMD is much more useful here

Response: Thank you for your positive comment on the standardized mean difference in Table 1.

Fig 2 This is somewhat confusing. I think it might be better as two figures (unless you are trying to encourage comparison of the main and secondary analysis - but why would that be good?)

Response: Old Fig 2 has been divided into two figures (new Fig 2 and 3) to avoid confusion.

Fig 4 Stacked bar charts are not a good graphic. Better to use a Cleveland dot plot

Response: Old Fig 4 (new Fig 5) has been revised using a Cleveland dot plot according to your recommendation.

Peter Flom

Thank you for your professional opinion and reasonable comments on the manuscript.

-----------------------------------------------------------

Reviewer #2: In this manuscript the author describes his retrospective cohort study done to assess the validity of the U.S.-derived Pooled Cohort Equations as a tool to estimate ASCVD risk reduction and total mortality benefit of statin therapy in a large Korean primary prevention population, 40-79 years of age, derived from the Korean National Health Information Database. ASCVD event rates and all-cause mortality in 58,265 individuals who initiated statins during 2007-2010 were compared with that in a similar number of non-users matched on propensity scores, from January 1, 2012 through December 31, 2017. A simulation was also performed for the population- based cohort of 659,759 adults. The authors used 3 tertiles of statin prescription refill data (regular, intermittent and occasional use) and correlated these with outcomes in groups at 3 levels of estimated risk (<5%, 5-9.9% and ≥10% 10-year risk). They concluded that across the spectrum of predicted ASCVD risk,

relative risk reduction with statin therapy was similar and was a function of estimated statin use. Because of the low absolute risk reduction in those at low risk, there appears to be minimal rationale for statin use in such individuals. However, in those with 10 year risk of 5% or greater, the study suggests that statin therapy is a reasonable population-based strategy for ASCVD risk and total mortality reduction. The authors point out the increased risk for diabetes in statin users, a finding that is consistent with published meta-analyses, and a reduced risk for diagnosed dementia, a finding that has not gained widespread acceptance based on other available data.

Thank you for your careful review of the manuscript.

The major strength of the study is the size of the population studied, and demonstrates the great value of a national health care database to help to establish prevention-related health policy. The methodology used for this type of study appears sound and has important public health policy implications for a population of a different ethnicity than that from which the Pooled Cohort Equations were derived. The use of big data from other countries with national health care systems would be of great interest to determine whether similar or divergent findings were noted.

Response: Thank you for your positive comments on this study. The manuscript has been revised with respect to your comments.

The limitations of the study include its retrospective design, lack of information on which statins and the intensity of statins used, baseline and attained LDL-C levels and the assumption of medication use based on prescription refill data.

Response: I have revised the Methods and findings in the Abstract to include the key limitations.

The paper could be improved by providing additional discussion about the intended use of the Pooled Cohort Equations, which were designed to estimate 10-year (and in selected patients, 30-year) risk for fatal and non-fatal MI and stroke in U.S. Caucasians and Blacks. Although their use is well validated for risk prediction in these populations, their use for this purpose in other populations remains to be determined. The use of the Pooled Cohort Equations as a tool to determine statin benefit clearly requires additional study in both the US and in other countries.

Response: The Discussion of the manuscript has been revised to provide more information about the Pooled Cohort Equations.

One of the key limitations of the Pooled Cohort Equations is their ability to help clinicians to appropriately allocate statin therapy for those in borderline risk (5-7.4% 10-year risk) and intermediate risk (7.5-19.9% 10 year risk) categories. As was described by Nasir and colleagues in their analysis of a racially diverse cohort, the Multi-ethnic Study of Atherosclerosis, approximately 50% of those patients estimated to be at borderline or intermediate 10 -year ASCVD risk using the Pooled Cohort Equations have no coronary calcification on cardiac CT imaging, and as such, would be expected to derive minimal benefit from statin allocation (Nasir K et al. J Am Coll Cardiol 2015;66:1657-68). Thus, if statins were allocated to the "statin eligible population" as suggested by the authors of the current study, and if one were to extrapolate the results of Nasir's study to the Korean population, a large percentage of patients could potentially be given a preventive treatment with a very low likelihood of absolute risk reduction benefit and a very high number needed to treat to prevent one event. In addition such therapy would potentially expose such patients to unnecessary additional laboratory testing, unneeded medical visits and the potential for statin associated side effects.

Response: I agree with you on this point. In the 5th paragraph and Conclusions in the Discussion, I have described the limitations of the Pooled Cohort Equations along with the debatable benefit of statins in individuals at intermediate predicted risk.

Thank you for your insightful comments.

-----------------------------------------------------------

Reviewer #3: The author examined the associations of statin use with adverse outcomes across the CVD risk scores using a large prospective cohort study. There are several concerns, especially statistical analyses, to be addressed as summarized below.

1. The study sample, study baseline, etc. in the Method section need to be more clarified. The author mentioned the date of the baseline was Jan 1, 2012. Does every participant have the same baseline? Is there any information on the date of the examination? The author explained 150,072 with a medical history of heart disease, stroke, or cancer or with an eGFR<30 were excluded, and then 83877 died/developed CVD, cancer, ESKD before baseline. Is there a gap between them? Please make sure.

Response: This study was a retrospectively designed cohort study, and the study used a public database that collected data for general research purposes rather than specifically for this study, although the NHIS collected the data prospectively. I regret the confusion due to the absence of a clear statement, and the Title of the manuscript has been revised to include the study design.

In this study, study outcomes were identified from the baseline (January 1, 2012) through the end of study (December 31, 2017). Medication use statuses were determined at the baseline and then in each year during the follow-up. Other covariates were determined once, using 6-year records of health screenings from the date of cohort entry (January 1, 2006) to the baseline. To clarify those, I have created a new S5 Table in the Supplement.

For this analysis, health screening and reimbursement records were collected for all eligible participants. After reviewing health screening records to the baseline, the participants with a history of heart disease, stroke, or cancer or with an eGFR of <30 ml/min/1.73 m^2^ were excluded. Then, from the remaining 792,727 participants, 83,877 who died or suffered CVD, cancer, and ESKD before baseline were excluded after reviewing NHIS reimbursement records to the baseline.

2. Did the author calculate the propensity score (PS) based on the variables in 2012? If so, I am concerned that the probability of receiving statin was accounted for the information after initiating statin among statin-user. Also, is there any reason for having a 2-year gap between statin users and non-users?

Response: I acknowledge that the outcome risks assessed in observational studies could easily be influenced by reverse causality or residual confounding.

In this study, statin use status (rather than statin initiation) was the primary exposure of interest. As you pointed out, the propensity score was a probability that a person would be a recent user rather than be a new user at the baseline. Accordingly, the propensity score-matched cohort included the recent-user and non-user pairs with balanced confounders that incorporated the measures even after statin initiation. However, the balanced confounders did not incorporate the measures after baseline. I believe that the outcome events that occurred during the follow-up would not reversely influence the confounders or the statin use status.

As you mentioned, this analysis excluded the users who received statins in 2006. Past statin users might be more likely to have unmeasured risk factors compared with nonusers or recent users. To minimize residual confounding by unmeasured factors, the analysis excluded the users who received statins in the year of cohort entry. In addition, the users who initiated statins in the last year (2011) before baseline were also excluded, since their statin use status (regular, intermittent, or occasional) could not reliably be determined at the baseline. I have revised the 2nd paragraph in the Methods to provide the reasons for the exclusion.

3. I am concerned about the CVD risk score calculated by the average level of continuous variables. For example, for statin users, total cholesterol levels can be changed after statin initiation, which can influence CVD risk score. Is that possible that those who were high-risk group when initiating statin were categorized as the low- or moderate-risk group in the current analysis? If so, the low- or intermediate-group may reflect the beneficial effects of statin. Also, the author may be comparing a mixture of treated/untreated risk score on statin vs. persons not taking statin.

Response: As you pointed out, the CVD risk scores could be varied according to statin treatment if the scores were calculated using total cholesterols without account of statin use. However, in this analysis, the risk scores were calculated using untreated total cholesterol levels (or imputed levels if untreated levels were not available). I regret the absence of a detailed description, and have described how untreated total cholesterol records were separately collected (new S1 Text) and changed the term “Total cholesterol” to “Untreated total cholesterol” in the cells in Tables 1 and 2 to avoid confusion.

4. In this context, the risk calculator was not based on time-varying information. Also, for the categorical variable, the author used the information at baseline, although smoking status is likely changeable over time.

Response: As you mentioned, the CVD risk score was calculated once at baseline. Almost all covariates (except for age category) would have a potential to change over time. However, if a time-varying covariate included in Cox models was causally related to treatment effect (e.g., risk scores calculated using statin-treated total cholesterols), it might falsely attenuate the causal association between treatment and outcomes. Hence, this analysis included antihypertensive and antidiabetic use statuses, which were not causally related to statin treatment (i.e., not related to statin-treatment effects) but could change significantly with changes in statin use (e.g., compliance or noncompliance with medication), as time-varying covariates. I believe that, even if other covariates that were expected to change independently of statin use were entered as time-varying covariates (rather than as fixed covariates), the association between statins and outcomes would persist with no substantial change.

Minor

1. The author should clarify how other covariates (e.g., proteinuria, smoking, blood pressure, etc.) were collected and defined for the analysis.

Response: To clarify that, the manuscript has been revised to include information on the covariates (S1 Text).

2. The author should mention the justification of using revised PCE.

Response: Among commonly used CVD risk calculators, this study chose the revised Pooled Cohort Equations on account of several points. The Pooled Cohort Equations predict atherosclerotic CVD events which can be prevented by statin therapy. Additionally, the calculator was developed in several prospective cohorts and recently updated to improve accuracy (reduce overestimation). I have revised the manuscript to mention the advantages and disadvantages of the calculator in the 5th paragraph in the Discussion.

3. Similarly, are there any justifications of secondary outcomes?

Response: This study identified secondary outcomes using reimbursement record-retrieved information on medications, surgeries, and in-hospital procedures, along with the diagnosis codes. Although there have been few publications validating such identifications yet, these may be more reliable than those using the diagnosis codes only. I have revised the Outcomes subsection in the Methods to describe these more detailedly.

Thank you for your helpful comments on the manuscript.

-----------------------------------------------------------

**References**

1.	Anderson AH, Yang W, Townsend RR, Pan Q, Chertow GM, Kusek JW, et al. Time-updated systolic blood pressure and the progression of chronic kidney disease: a cohort study. Ann Intern Med. 2015;162: 258–265. doi:10.7326/M14-0488

2.	ACC/AHA ASCVD Risk Calculator. [cited 22 Aug 2020]. Available: http://www.cvriskcalculator.com/
